# Association of nutritional glycaemic indices with global DNA methylation patterns: results from the Moli-sani cohort

**DOI:** 10.1186/s13148-022-01407-3

**Published:** 2022-12-28

**Authors:** Fabrizia Noro, Federica Santonastaso, Annalisa Marotta, Marialaura Bonaccio, Sabatino Orlandi, Alfonsina Tirozzi, Simona Costanzo, Amalia De Curtis, Francesco Gianfagna, Augusto Di Castelnuovo, Furio Brighenti, Chiara Cerletti, Maria Benedetta Donati, Giovanni de Gaetano, Licia Iacoviello, Alessandro Gialluisi, Benedetta Izzi, for the Moli-sani Study Investigators, Licia Iacoviello, Giovanni de Gaetano, Maria Benedetta Donati, Marialaura Bonaccio, Americo Bonanni, Chiara Cerletti, Simona Costanzo, Amalia De Curtis, Augusto Di Castelnuovo, Alessandro Gialluisi, Francesco Gianfagna, Mariarosaria Persichillo, Teresa Di Prospero, Jos Vermylen, Renzo Pegoraro, Antonio Spagnolo, Deodato Assanelli, Livia Rago, Simona Costanzo, Marco Olivieri, Teresa Panzera, Augusto Di Castelnuovo, Marialaura Bonaccio, Simona Costanzo, Simona Esposito, Alessandro Gialluisi, Francesco Gianfagna, Sabatino Orlandi, Emilia Ruggiero, Alfonsina Tirozzi, Amalia De Curtis, Sara Magnacca, Fabrizia Noro, Alfonsina Tirozzi, Mariarosaria Persichillo, Francesca Bracone, Teresa Panzera, Americo Bonanni

**Affiliations:** 1 Department of Epidemiology and Prevention, IRCCS Neuromed, Via Dell’Elettronica, 86077 Pozzilli, IS Italy; 2 EPIMED Research Center, Department of Medicine and Surgery, University of Insubria, Varese, Italy; 3 Mediterranea Cardiocentro, Naples, Italy; 4 Department of Food and Drug, University of Parma, Parma, Italy; 5 Present Address: Human Technopole, Viale Rita Levi Montalcini 1, 20157 Milan, Italy; 6 Present Address: European School of Molecular Medicine, University of Milan, 20122 Milan, Italy; 7 Present Address: Center of Predictive Molecular Medicine, Center for Excellence on Ageing and Translational Medicine (CAST), University of Chieti-Pescara, Chieti, Italy

**Keywords:** Methylation, Hydroxymethylation, Glycaemic index, Glycaemic load, Metabolic disorders

## Abstract

**Background:**

High dietary glycaemic index (GI) and load (GL) have been associated with increased risk of various cardiometabolic conditions. Among the molecular potential mechanisms underlying this relationship, DNA methylation has been studied, but a direct link between high GI and/or GL of diet and global DNA methylation levels has not been proved yet. We analyzed the associations between GI and GL and global DNA methylation patterns within an Italian population.

**Results:**

Genomic DNA methylation (5mC) and hydroxymethylation (5hmC) levels were measured in 1080 buffy coat samples from participants of the Moli-sani study (mean(SD) = 54.9(11.5) years; 52% women) via ELISA. A 188-item Food Frequency Questionnaire was used to assess food intake and dietary GI and GL for each participant were calculated. Multiple linear regressions were used to investigate the associations between dietary GI and GL and global 5mC and 5hmC levels, as well as the proportion of effect explained by metabolic and inflammatory markers. We found negative associations of GI with both 5mC (*β* (SE) = − 0.073 (0.027), *p* = 0.007) and 5hmC (− 0.084 (0.030), *p* = 0.006), and of GL with 5mC (− 0.14 (0.060), *p* = 0.014). Circulating biomarkers did not explain the above-mentioned associations. Gender interaction analyses revealed a significant association of the gender-x-GL interaction with 5mC levels, with men showing an inverse association three times as negative as in women (interaction *β* (SE) = − 0.16 (0.06), *p* = 0.005).

**Conclusions:**

Our findings suggest that global DNA methylation and hydroxymethylation patterns represent a biomarker of carbohydrate intake. Based on the differential association of GL with 5mC between men and women, further gender-based separate approaches are warranted.

**Supplementary Information:**

The online version contains supplementary material available at 10.1186/s13148-022-01407-3.

## Background

Excessive intake of carbohydrates favoring higher glucose levels has been extensively linked to clinical outcomes including cardiometabolic diseases [1] and neurological disorders [2]. Indeed, increasing evidence in population studies supports a strong relationship between quality and quantity of carbohydrates ingested with foods and increased risk of certain types of cancers [3, 4], diabetes [5, 6] and cardiovascular diseases [7–9]. This is likely explained through carbohydrates’ direct implications in regulating blood glucose levels, as well as in changing postprandial hormonal and metabolic responses in humans [10]. For these reasons, increasing attention has been given in the last decades to control the quantity and quality of carbohydrates’ dietary intake in clinical practice, through specific indices.

Among these, glycaemic index (GI) and glycaemic load (GL) represent two common dietary indices increasingly used to measure and control carbohydrate intake in people affected by cardiometabolic disorders, like diabetes [11]. GI [12], generally calculated from International GI tables [13, 14], is a measure of carbohydrate quality and refers to the postprandial blood glucose increase in response to a given carbohydrate intake, when compared with a reference food (either glucose or white bread). GL represents instead an indicator of both quality and quantity of carbohydrates and is calculated by multiplying the GI of a food item with the available carbohydrate content [11]. While both higher GI and higher GL have been associated with increased disease risk [3–9], their relationship with potential molecular mechanisms underlying cardiometabolic dysfunction remains largely neglected.

Altered DNA methylation patterns, particularly the level of methylation (5mC) and hydroxymethylation (5hmC) in the genome, have been often associated with type 2 diabetes (T2D) and with a general oxidative stress status [15–18].

Investigations focusing on the relationship between DNA methylation patterns and dietary GI and GL mainly consist of interventional studies on maternal dietary habits, aimed at detecting methylation changes in the offspring [19]. Geraghty et al. [20] analyzed DNA methylation levels at 771,484 CpGs sites across the genome in free DNA from cord blood serum in 60 newborns involved in the ROLO study. The methylome of low GI intervention newborns was found to be significantly lower than in controls [20]. By comparing two groups of pregnant women following a reduced vs. an increased carbohydrate diet, Yan et al. observed specific placenta DNA methylation changes at genes involved in insulin regulation, namely *PLIN1*, *CPT1B*, *SSTR4* and *CIDEA* [21]. In a recent observational study, Alick et al. [22] found out that a maternal periconceptional diet characterized by a high glycaemic loading was associated with poorer neurodevelopmental status of children, in particular anxiety-related behavior, and with an increased mean methylation level of the imprint control region of SGCE/PEG10.

In spite of these suggestive independent lines of evidence, we are not aware of any study so far investigating the relationship between global methylation patterns and glycaemic index and load of diet, assessing both measures in the same subjects. Such a design would help in (1) building a closer relationship between nutritional and genome methylation patterns and (2) identifying the potential implications of dietary habits, which are of remarkable importance for cardiometabolic patients, on novel epigenetic measures.

In the present study, we aimed at investigating the associations between both GI and GL and global DNA methylation within an Italian population cohort enrolled in the Moli-sani study. We already reported a fine-grained analysis of global DNA methylation patterns at different nutritional levels in the same cohort, identifying a direct relationship between daily intake of zinc and global DNA methylation [23]. Here, we focused on dietary glycaemic indices to build a bridge with altered epigenetic patterns potentially underlying metabolic conditions.

## Results

The characteristics of the analyzed sub-cohort (*N* = 1080 with at least an epigenetic measure available) are summarized elsewhere [23] and in Table 1. Compared to the whole Moli-sani cohort, the population under study showed similar sex ratio (48.0% vs 48.1% men) but slightly lower age (mean (SD) age 54.9 (11.5) year vs 55.8 (12.0) years, *p* < 0.0001), due to the removal of prevalent CVD cases. Similarly, in the analyzed sub-cohort there was a lower prevalence of diabetes (3.6% vs 5.0%, *p* = 0.02) and hyperlipidemia (4.2% vs 7.9%), as well as a higher energy intake (2210.19 kcal/d vs 2079.01 kcal/d, *p* < 0.0001). Overall, there was no systematic difference between the analyzed sub-cohort and the whole Moli-sani population, except those due to removal of individuals with history of CVD.

**Table 1:** Baseline characteristics of the analyzed sub-cohort (*N* = 1080) compared to the whole Moli-sani cohort (*N* = 24,325)

Variable	Sub-cohort			Whole Moli-sani cohort		
	*N*	Mean	SD	*N*	Mean	SD
Age (years)	1080	54.91	11.52	24,325	55.79	11.96
MDS	1080	4.73	1.6	24,221	4.35	1.64
Leisure time physical activity (met-h/d)	1080	3.6	4.03	24,325	3.48	4.02
BMI (kg/m^2^)	1079	28.04	4.54	24,308	28.06	4.78
Energy intake (Kcal/d)	1080	2210.19	682.57	24,225	2079.01	667.66
Abdominal Obesity (WHR)	1079	0.92	0.07	24,297	0.92	0.08
Monocytes (%)	1037	5.93	2.04	23,544	7.09	2.12
Granulocytes (%)	1037	60.69	7.68	23,542	60.25	7.82
Lymphocytes (%)	1037	33.33	7.39	23,545	32.63	7.34
Glucose (mg/dL)	1075	100.01	25.52	23,099	101.55	25.39
C-Peptide (ng/mL)	1040	1.39	0.61	22,379	1.78	0.84
Insulin (pmoli/L)	1046	49.81	29.29	22,458	60.49	44.63
Glycemic index	1080	54.26	3.05	23,178	54.32	3.19
Glycemic load	1080	140.95	51.47	23,178	131.42	50.52

Categorical variables	N	n	%	N	n	%
Males (n. %)	1080	518	47.96	24,325	11.702	48.11
*Education*						
Primary	1080	223	20.65	24,286	6.268	25.81
Lower secondary	1080	285	26.39	24,286	6.742	27.76
Upper secondary	1080	405	37.5	24,286	8.259	34.01
Post-secondary	1080	167	15.46	24,286	3.017	12.42
*Health conditions*						
CVD	1068	0	0	24,023	1.427	5.94
Cancer	1076	35	3.25	24,198	788	3.26
Diabetes	1065	38	3.57	24,017	1.214	5.05
Metformine	1065	31	2.91	24,017	803	3.34
Hyperlipidaemia	1061	45	4.24	24,092	1.911	7.93
*Drinking status (drinkers)*						
Ever	1080	151	13.98	24,325	6.156	25.31
Current	1080	774	71.67	24,325	14.650	60.23
Former	1080	96	8.89	24,325	1.032	4.24
Occasional	1080	57	5.28	24,325	1.515	6.23
Missing	1080	2	0.19	24,325	972	4
*Smoker status (smokers)*						
Ever	1078	527	48.89	24,296	12.050	49.6
Current	1078	263	24.4	24,296	5.582	22.97
Former	1078	288	26.72	24,296	6.664	27.43

*MDS* Mediterranean diet score, *BMI* body mass index, *WHR* waist to hip ratio, *CVD* cardiovascular disease

Association analyses of glycaemic parameters in the analyzed sub-cohort (Table 2) revealed significant negative associations of GI with both 5mC (standardized *β* (Standard Error) = − 0.073 (0.027), *p* = 0.007) and 5hmC (*β* (SE) = − 0.084 (0.030), *p* = 0.006) measured on buffy coat samples from the studied subjects. A significant negative association was also observed between GL and 5mC (*β* (SE) = − 0.146 (0.060), *p* = 0.015), but not with 5hmC. These associations remained significant after additional adjustments for other potential confounding factors like use of metformin (Additional file 1: Table S1).

**Table 2:** Association of dietary glycaemic parameters with methylation (5mC) and hydroxymethylation (5hmC) measures

Glycaemic parameter	Epigenetic marker	Beta	SE	T-stat	*P* value
Glycaemic load	5mC	− 0.146	0.060	− 2.441	0.015
Glycaemic index	5mC	− 0.073	0.027	− 2.699	0.007
Glycaemic load	5hmC	− 0.028	0.068	− 0.412	0.680
Glycaemic index	5hmC	− 0.084	0.030	− 2.77	0.006

These associations were adjusted for age, sex and educational attainment, white blood cell fractions, smoking, leisure time physical activity, abdominal obesity, alcohol intake, prevalent diabetes, hyperlipidaemia and cancer

In the mediation analysis of different circulating biomarkers—including C-reactive protein (CRP), glucose, C-peptide, insulin, total cholesterol, LDL, HDL—no significant proportion of the above- mentioned associations was explained by any of the circulating markers tested (Table 3).

**Table 3:** Mediation analysis of metabolic parameters on the association between dietary glycaemic parameters and methylation/hydroxymethylation measures

Potential mediator	5mC versus GI	5hmC versus GI	5mC versus GL
Glucose	− 0.017 [− 0.119; 0.020], 0.37	− 0.007 [− 0.082; 0.020], 0.52	0.060 [− 0.014; 0.330], 0.10
C-peptide	0.002 [− 0.030; 0.050], 0.74	0.010 [− 0.039; 0.080], 0.56	1.69e−03 [− 4.97e−02; 0.06], 0.80
Insulin	− 3.32e−04 [− 3.87e−02; 0.03], 0.94	0.002 [− 0.026; 0.05], 0.77	9.30e−04 [− 4.99e−02; 0.07], 0.86
Total cholesterol	0.0002 [− 0.0308; 0.03], 0.93	7.02e−05 [− 2.66e−02; 0.03], 0.97	− 8.22e−03 [− 1.53e−01; 0.07], 0.65
LDL	0.002 [− 0.027; 0.06], 0.75	− 0.006 [− 0.076; 0.03], 0.68	− 1.06e−04 [− 3.95e−02; 0.04], 0.98
HDL	4.46e−04 [− 3.29e−02; 0.04], 0.90	3.91e−05 [− 2.63e−02; 0.03], 0.98	1.41e−03 [− 9.13e−02; 0.12], 0.93
CRP (log-scale)	5.69e−04 [− 3.45e−02; 0.04], 0.88	9.63e−04 [− 3.22e−02; 0.04], 0.83	− 2.48e−04 [− 5.30e−02; 0.04], 0.93

Proportion of the association mediated (along with 95% Confidence Interval in squared brackets) and relevant *p* value is reported for each potential mediator tested. *Note:* since the proportion of mediated effect is computed as the proportion of average causal mediation effect (ACME) over total effect of the exposure on the outcome (TE), this may have also negative values when ACME and TE are not concordant. This scenario suggests no proportions of the association are mediated, as further supported by *p* values

*LDL* low-density lipoprotein, *HDL* high-density lipoprotein, *CRP* C-reactive protein, *GL* Glycaemic Load, *GI* Glycaemic Index

Gender interaction analyses revealed a significant association of the interaction term between gender and glycaemic load with 5mC levels (interaction *β* (SE) = − 0.16 (0.06), *p* = 0.005), with men showing an inverse association more than three times as large as in women (Table 4). No other significant interactive associations were detected.

**Table 4:** Gender interaction associations of dietary glycaemic parameters with 5mC and 5hmC

Epigenetic signature	Exposure	Beta (SE) interaction	*p* for interaction	Beta (SE) women
5mC	GL	–	–	− 0.042 (0.070)
	GL * gender (men)	− 0.159 (0.057)	0.005	–
	GI	–	–	− 0.044 (0.036)
	GI * gender (men)	− 0.068 (0.055)	0.216	–
5hmC	GL	–	–	0.037 (0.080)
	GL * gender (men)	− 0.099 (0.064)	0.123	–
	GI	–	–	− 0.092 (0.040)
	GI * gender (men)	0.017 (0.061)	0.78	–

Association of dietary glycaemic parameters and their interaction terms with gender with 5mC and 5hmC. *Note:* as per interaction analysis output, we report Beta (SE) and p of the index-by-gender (men) interaction term, as well as the Beta (SE) values of the association in women. Associations Beta values in men can be computed by summing the two Betas

*GL* Glycaemic Load, *GI* Glycaemic Index

## Discussion

Here we report, for the first time, a concordant negative association between two measurements reflecting carbohydrates’ quality and quantity (GI and GL), and global methylation levels measured within the same subjects from a general population. These associations resisted correction for several factors influencing hypomethylation, including use of metformin [24, 25] or self-reported diabetic status.

Nutritional factors, among all the environmental stimuli, can affect epigenetics both transiently and chronically [26–28]. The specific epigenetic changes caused by sustained hyperglycaemia are the basis for the establishment of the so-called metabolic memory [29] and are the means by which exposure to high glucose exerts its long-lasting detrimental effects on human health in the context of cancer [30], diabetes [31–34] and CVD [35–37]. Global hypomethylation is a generally accepted hallmark of cancer [38]. More controversial are the published studies that consider global DNA methylation levels and CVD or diabetes. However, lower global DNA methylation (5mC), evaluated with a similar technique to the one used in this study, has been generally associated with clinical and subclinical CVD phenotypes including hypertension, atherosclerosis, coronary artery disease and increased CVD risk in postmenopausal women [38–42]. Very few studies have investigated global DNA methylation in the context of diabetes and metabolic syndrome, generally reporting lower 5mC and 5hmC levels [16, 17] although not always consistently [18].

Consuming high GL foods is known to cause an increase in blood glucose and insulin levels with consequent increase in plasma-free fatty acids [43]. All together these factors contribute to lower insulin sensitivity and to the development of dyslipidemia [10, 43, 44]. Though some controversial data have been published on the topic, several observational studies have supported these findings by identifying a direct association between dietary GL and glucose metabolism parameters [45–52]. Based on this, GI and GL have been extensively considered and validated as risk factors for chronic diseases [3–9]. Therefore, identifying a possible molecular mechanism linking these variables to health outcomes could bare important opportunities to identify a novel marker for clinical risk assessment.

Higher GI and GL in the studied Moli-sani sub-cohort were associated with lower global methylation and hydroxymethylation levels in the genome. Although to our knowledge there are no such comparable studies in the field, we evaluated our findings in relation to previous studies on the link between T2D and genomic methylation patterns. Indeed, our observations are partly concordant with previous epidemiological evidence of lower hydroxymethylation levels found in diabetic patients compared to controls and with the functional evidence that glucose treatment increases 5hmC levels in specific cell lines like PBMCs, HUVECs and TF-1 [16]. This mechanism is mediated by downregulation of TET2 (ten-eleven translocation 2 protein) levels, an enzyme involved in the conversion of 5-methylcytosine into 5-hydroxymethylcytosine in the genome. This effect is counterbalanced by metformin treatment, which increases TET2 stability and 5hmC levels [16]. Our findings are also in line with previous reports of decreased 5mC levels in T2D patients compared to controls, also after adjustment for use of metformin. Conversely, contrasting evidence of higher 5mC and 5hmC levels in peripheral blood cells of poorly controlled compared to well-controlled diabetic patients and healthy controls has been reported [18]. Moreover, the lack of evidence of mediation by glucose, insulin and C-peptide in the link between dietary glycaemic indices and methylation patterns suggests that other biomarkers should be investigated to explain the significant associations observed here. Therefore, further studies aimed at disentangling the link between dietary glycaemic parameters and altered genomic methylation are needed to clarify the mechanisms linking nutrition, methylation patterns and diabetes-related traits.

Another interesting finding in our study was that some of the significant associations observed were not concordant between genders. Prominently, the decrease in methylation levels per SD increase of glycaemic load was more than three times as negative in men compared to women. If supported by independent studies, these findings may open a gender-based perspective on the investigation of potential effects of dietary glycaemic quantity, with immediate translational implications for the control of carbohydrate intake in patients also based on their gender. In view of the known link between metabolic and cardiovascular diseases [7–9], 5hmC could represent a specific novel marker for cardiometabolic risk prediction in women.

## Limitations and implications for future studies

Although this study has the merit to provide a contribution to the molecular epidemiology of the relationship between DNA methylation and glucose-related dietary patterns, an aspect very scarcely investigated yet in the field of nutrigenomics, we need to acknowledge some limitations of the present work. The FFQ used in this study was not specifically designed to evaluate dietary GI and GL, but to provide estimates of total carbohydrate and total energy intake. Furthermore, GI and GL estimates derived from FFQs may not take into account several factors that can influence the postprandial glycaemic response, such as varying meal frequency, varying cooking methods or chewing habits. Also, dietary data were self-reported and this may lead to recall bias. Similarly, the cross-sectional setting does not allow to establish clear directionality of effect between glycaemic parameters and epigenetic modifications, nor does it give any precise information linking glycaemic nutritional parameters, altered methylation patterns and chronic disease risk. Also, the possibility of residual confounding by unmeasured factors cannot be fully excluded. This—along with the low number of T2D cases (< 40) in the analyzed sub-cohort—did not allow to test potential mediation effects of methylation patterns in the link between dietary parameters and diabetes, which was instead used as a covariate. Finally, our global measure of DNA methylation/hydroxymethylation does not allow to further explore potential mechanisms linking a specific gene or pathway to quality and quantity of carbohydrate intake, which makes it difficult to understand the functional meaning of these associations.

## Conclusions

This study represents, to the best of our knowledge, the first attempt to investigate the relationship between methylation patterns in the genome and dietary glycaemic parameters in the same individual from an adult population cohort. Our findings suggest that global DNA methylation and hydroxymethylation patterns can be used as biomarkers of carbohydrate intake. Further approaches are necessary to better understand the gender-based potential effects of dietary GI and GL.

## Methods

### Study population

The study population was already described in Noro et al. [23] and composed of a randomly selected sub-cohort of 1,160 participants of the Moli-sani study (*N* = 24,325; 49.20% men; age ≥ 35 years, recruited between 2005 and 2010) [53, 54]. Subjects with incomplete dietary questionnaires or with missing values in the studied variables were excluded from the analysis to a final number of 1080 subjects.

### DNA extraction and global DNA methylation assessment

We used a silica matrix-based method to extract buffy coat DNA as described in [55]. Out of the original 1160 DNA samples, 1140 were selected based on their DNA quality to be further used in the methylation study (see below). We used the MethylFlash Global DNA Methylation (5mC) ELISA Easy Kit (colorimetric) and the MethylFlash Hydroxymethylated DNA 5hmC Quantification Kit (colorimetric) (EpiGentek), according to the manufacturer’s instructions, to assess global levels of 5-methylcytosine (5mC) and 5-hydroxymethylcytosine (5hmC), respectively. DNA methylation quality control and statistical analyses were performed using R (The R Project, 2020; https://www.r-project.org/).

We overall measured 5mC and 5hmC global levels for 1214 samples (including 1,140 original and 74 duplicate samples). Of these, samples with absorbance optical density (OD) values below the mean of negative controls plus 2 standard deviations (SDs) for both global methylation measurements were set to missing as described [23]. We additionally excluded: (1) 17 and 2 outlier samples for 5mC and 5hmC, respectively, defined as samples with absolute values of standardized methylation levels above 3 standard deviations; and (2) all prevalent CVD cases (56 and 58 samples for 5mC and 5hmC, respectively) to exclude reverse causality of CVD on methylation levels [56]. Finally, 1067 samples for 5mC and 1075 samples for 5hmC were used in the following statistical analyses. 5mC and 5hmC showed a modest but significant inverse correlation (Pearson’s *r* = − 0.21, *p* = 1.1 × 10^–11^). For both 5mC and 5hmC, the study population distributions were approaching normality (Additional file 1: Figure S1a, b).

### Dietary assessment and calculation of dietary glycaemic index and load

Food intake during the year before enrollment was assessed by the Italian version of the semiquantitative EPIC food frequency questionnaire (FFQ) [57]. The FFQ contains 14 sections (i.e., pasta/rice, soup, meat (excluding salami and other cured meats), fish, raw vegetables, cooked vegetables, eggs, sandwiches, salami and other cured meats, cheese, fruit, bread/wine, milk/coffee/cakes and herbs/spices) with 248 questions concerning 188 different food items.

Frequencies and quantities of each food were linked to Italian Food Tables [58] using a specifically designed software in order to obtain estimates of daily intake of macro- and micronutrients plus energy.

The average dietary GI for each volunteer was calculated as the sum of the GIs of each food item consumed, multiplied by the average daily amount consumed and the percentage of carbohydrate content, all divided by the total daily carbohydrate intake. The GL was calculated similarly except that there was no division by total carbohydrate intake.

Generally, the more digestible a carbohydrate is, the higher its GI will be. Some carbohydrates are absorbed quickly and lead to rapid rise in blood glucose (high GI), while others release glucose more slowly (low GI).

The glycaemic load (GL), instead, was calculated by multiplying the GI of each specific food for its total carbohydrate content (g), then dividing by 100 [59]. GL is therefore meant to represent the actual increase of blood glucose caused by ingesting a given quantity of carbohydrates contained in a portion of food.

Dietary GI and GL for each study participant were calculated as the sum of the GIs and GLs of all foods consumed in the diet [60]. GI and GL showed a modest positive correlation (*r* = 0.13, *p* = 1.3 × 10^–5^) and were their distributions approached normality in the population under study (Additional file 1: Figure S2a, b).

### Covariates assessment and selection

Several covariates were considered or tested as potential confounders of the relationship between glycaemic index (GI) and load (GL) and global DNA methylation levels (5mC/5hmC).

Among them, *sex* and *age* were selected by default, since they are typical confounding factors which influence both methylation measures [61, 62] and nutritional patterns [63]. Similarly, energy intake (kcal/day) was included because also the amount of eaten food might influence participant methylation levels [64], as was e*ducational attainment* (defined as completed school level: primary, lower, upper secondary and post-secondary), which is associated with both methylation [65] and nutritional patterns [66]. *White blood cell* (granulocytes, monocytes, lymphocytes) fractions were also included by default to account for their heterogeneity, since global DNA methylation was measured on DNA extracted from these cells.

Other variables—including *smoking habits*, *leisure time physical activity*, *abdominal obesity, alcohol intake*, *diabetes*, *hyperlipidaemia* and *cancer* (as defined previously)—were added to the models since they showed a univariate trend of association with both the nutritional exposure and the methylation outcome (*p* < 0.2). The definition of covariates is reported in Additional file 1.

### Statistical analyses

Statistical analyses were carried out in R (https://www.r-project.org/). The association between GI and GL of diet (exposures) and standardized global methylation levels (outcome) was analyzed through linear regressions (*lm()* function in R), separately for 5mC and 5hmC, adjusting for different potential confounders of these relationship, which included age, sex and educational attainment, white blood cell fractions, smoking, leisure time physical activity, abdominal obesity, alcohol intake, prevalent diabetes, hyperlipidaemia and cancer (see Supplementary Methods for definitions and details on selection).

Sensitivity analyses were carried out to disentangle significant associations detected, through further adjusting them for use of metformin—an antidiabetic drug with known altering effects on DNA methylation (24)—so as to ensure that the detected associations were independent from this factor.

Moreover, we performed mediation analyses through the *mediate* function of the *mediation* package (https://cran.r-project.org/web/packages/mediation/), to estimate the proportion of association between dietary glycaemic indices (GI and GL) and methylation measures (5mC/5hmC) explained by different circulating biomarkers, including high sensitivity C-reactive protein (CRP, tagging circulating inflammation), glucose, insulin and C-peptide levels (tagging glucose homeostasis) and total, LDL and HDL cholesterol.

Similarly, we performed gender interaction association analyses for both exposures and both outcomes mentioned above, to detect potential gender-specific associations.

## Supplementary Information

**Additional file 1**. Definition of covariates. Definition of all the covariates used in the study. **Table S1**. Sensitivity analyses of glycaemic dietary parameters with methylation and hydroxymethylation measures.

## Data Availability

The data underlying this article will be shared on reasonable request to the corresponding author. The data are stored in an institutional repository (https://repository.neuromed.it) and access is restricted by the ethical approvals and the legislation of the European Union.

## Abbreviations

5mC: 5-Methylcitosine
5hmC: 5-Hydroxymethylcitosine
CRP: C-reactive protein
CVD: Cardiovascular disease
FFQ: Food frequency questionnaire
GI: Glycaemic index
GL: Glycaemic load
HDL: High-density lipoprotein
LDL: Low-density lipoprotein
MDS: Mediterranean Diet Score
OD: Optical density
SD: Standard deviation
T2D: Type 2 diabetes
TET: Ten-eleven translocation protein
