# Supplementary material for: Association of nutritional glycaemic indices with global DNA methylation patterns: results from the Moli-sani cohort
Source: Clin Epigenetics. 2022 Dec 28;14:189. doi: 10.1186/s13148-022-01407-3 (PMC9798643; doi:10.1186/s13148-022-01407-3)
Supplement: Supplementary file 1 — Additional file 1. Definition of covariates. Definition of all the covariates used in the study. Table S1. Sensitivity analyses of glycaemic dietary parameters with methylation and hydroxymethylation measures. [file 13148_2022_1407_MOESM1_ESM.docx]

**Additional file 1**

***Definition of covariates***

All the variables explained below were used in the analysis as continuous variables, unless otherwise stated.

For e*ducational attainment*, subjects were divided into four categories, based on the school level completed: *None/Primary, Lower secondary, Upper secondary* and *Post-secondary*.

For smoking status, subjects were assigned to three categories based on their cigarette smoking habits: ever, current and former.

Leisure-time physical activity was assessed through a structured questionnaire and expressed as daily energy expenditure in metabolic equivalent task-hours (MET-h/day) (Ainsworth et al., 2000).

Food intake was assessed by the validated Italian EPIC food frequency questionnaire. The EPIC questionnaire also allowed to compute the daily *energy* (Kcal/day) and *alcohol intake* (g/day) for the subjects assessed.

Alcohol users were classified into current, former (since more than 1 year), occasional and never drinkers, with subjects presenting missing information or not willing to answer classified as an additional (dummy) class.

Prevalent *diabetes*, *and* *hyperlipidaemia* were defined as dichotomous variables (Yes/No), based on the reported and verified use of specific drugs for the treatment of these disorders, while prevalent *cancer* classification was based merely on self-report of medical history of the disease (possibly supported by medical documentation or by the use of specific drugs).

Metformin use was assessed by questionnaire collected during the recruitment and providing accurate information on the use (frequency, dose, compliance) of the medication. The questionnaire on drug usage was directly linked to the Italian National drug index.

Waist circumference (cm) was measured in the middle between the 12^th^ rib and the iliac crest, while hip circumference (cm) was measured around the buttocks. Waist-to-hip ratio (WHR) was calculated, and the resulting measure of *abdominal obesity* was inferred as a dichotomous variable (Yes/No), defining as *abdominal* obese men with WHR ≥ 0.90 and women with WHR ≥ 0.85 (World Health Organization., 2011).

Lipids (total cholesterol, HDL-cholesterol, triglycerides) and blood glucose were assayed in serum samples by enzymatic reaction methods using an automatic analyzer (ILab 350, Instrumentation Laboratory, Milan, Italy) and quality control for lipids and glucose was obtained by two commercial standards SeraChem® 1 (a control for normal levels) and SeraChem® 2 (a control for pathological high levels); the coefficients of variability (CV) of these two commercial standards were respectively 4.9% and 5.2% for blood total cholesterol; 3.2% and 3% for HDL-cholesterol; 5.2% and 5.3% for triglycerides, 4.7% and 4.1% for blood glucose.

C-peptide and insulin levels were measured from frozen plasma/serum samples (≥4 h fasting) using the Abbott Architect i2000 system (Abbott Laboratories, Chicago, Illinois, USA) as a part of the BiomarCare project in Hamburg, Germany. (<http://www.biomarcare.eu/>)

High sensitivity C-reactive protein (hs-CRP) was measured in fresh serum samples by a particle-enhanced immune-turbidimetric assay (ILab 350, Instrumentation Laboratory, Milan, Italy). Quality control for hs-CRP was maintained using in-house serum pool and commercial laboratory standard; inter-day coefficients of variability for CRP were 4.2% and 5.5%.

**Table S1.** Sensitivity analyses of glycaemic dietary parameters with methylation and hydroxymethylation measures.

| **Exposure** | **Outcome** | **Beta** | **SE** | **T-stat** | **P-value** |
| --- | --- | --- | --- | --- | --- |
| **Glycaemic index** | 5mC | -0.023 | 0.009 | -2.615 | 0.009 |
| **Glycaemic index** | 5hmC | -0.027 | 0.010 | -2.677 | 0.008 |
| **Glycaemic load** | 5mC | -0.002 | 0.001 | -2.131 | 0.030 |

We report Beta coefficients, the corresponding Standard Error (SE), T-statistics, and P-value from sensitivity analyses adjusting for the use of metformin, in addition to covariates included in the main regression models.

**Figure S1.** 5mC and 5hmC distributions.

**a)**

**
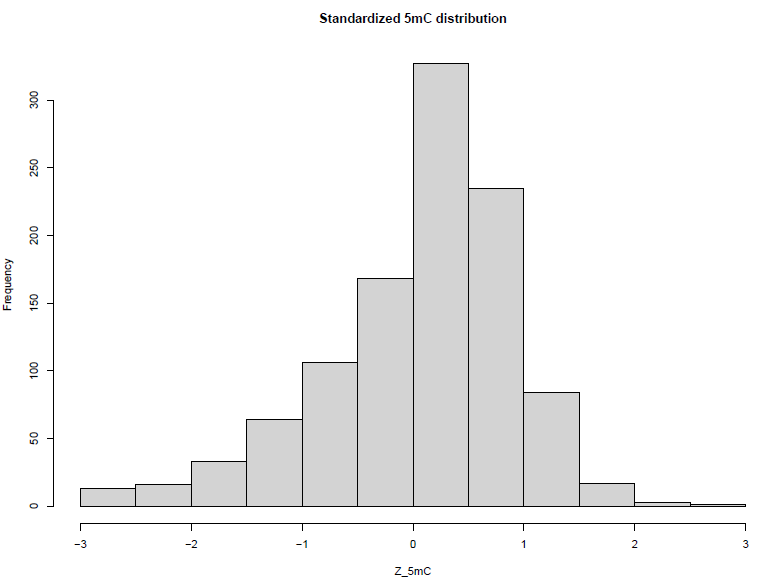
**

**b)**

**
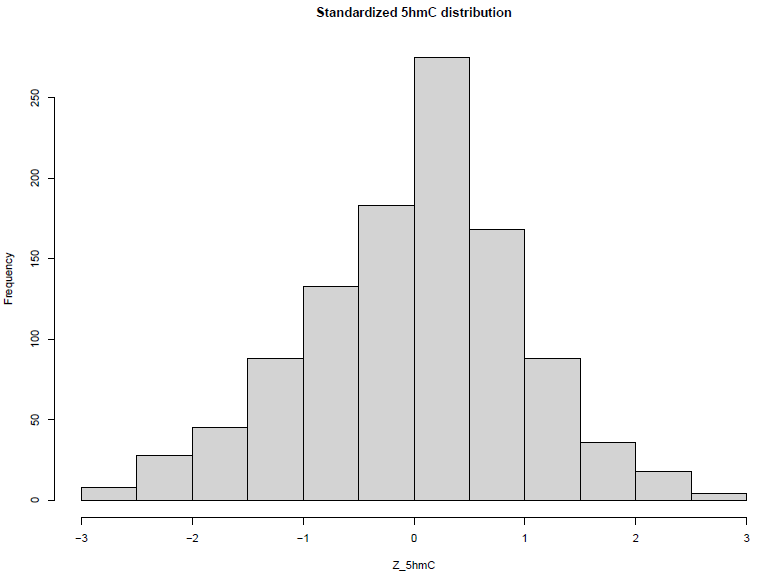
**

**Figure S2.** GI and GL distributions.

**a)**

**
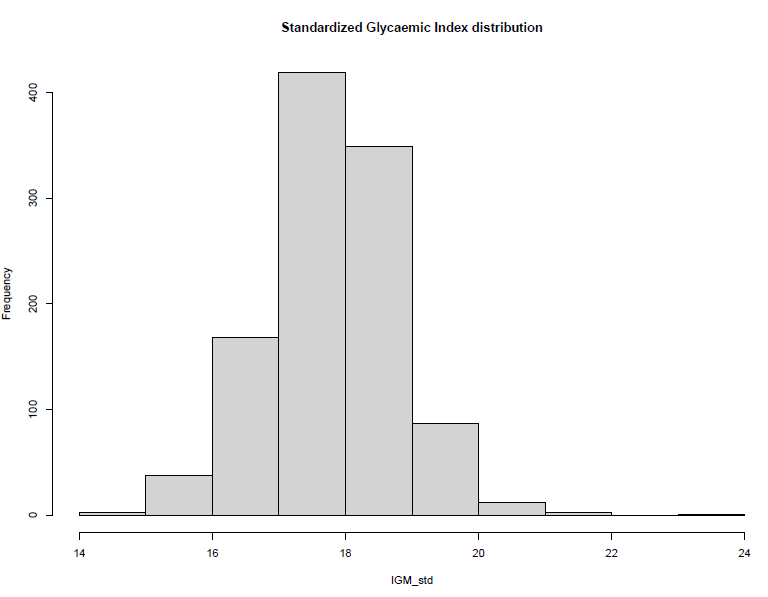
**

**b)**

**
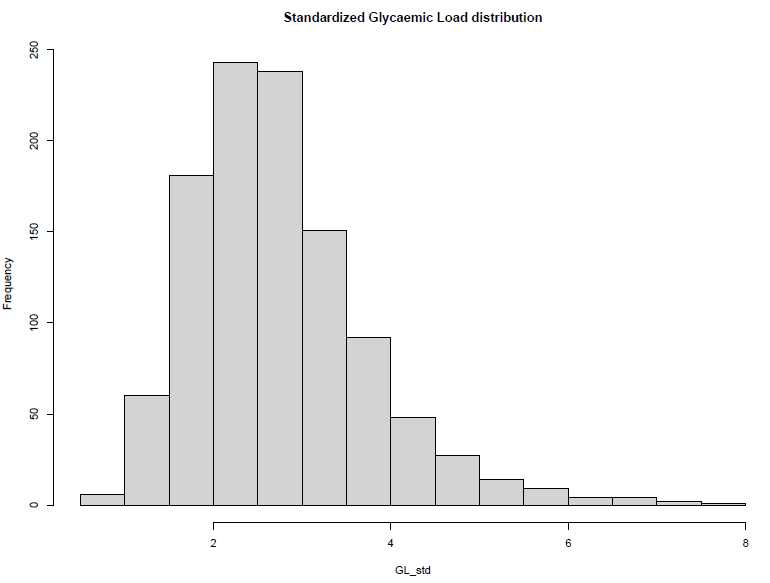
**
